# In vivo activation of pH-responsive oxidase-like graphitic nanozymes for selective killing of *Helicobacter pylori*

**DOI:** 10.1038/s41467-021-22286-x

**Published:** 2021-03-31

**Authors:** Lufeng Zhang, Liang Zhang, Hui Deng, Huan Li, Wentao Tang, Luyao Guan, Ye Qiu, Michael J. Donovan, Zhuo Chen, Weihong Tan

**Affiliations:** 1 Molecular Science and Biomedicine Laboratory, State Key Laboratory of Chemo/Bio-Sensing and Chemometrics, College of Chemistry and Chemical Engineering, College of Biology, Hunan Provincial Key Laboratory of Biomacromolecular Chemical Biology, Hunan University, Changsha, China; 2 Department of Gastroenterology, the Third Xiangya Hospital of Central South University, Changsha, China; 3 The Cancer Hospital of the University of Chinese Academy of Sciences, Institute of Basic Medicine and Cancer (IBMC), Chinese Academy of Sciences, Hangzhou, Zhejiang China

**Keywords:** Antimicrobials, Nanomedicine

## Abstract

*Helicobacter pylori* infection is a major etiological factor in gastric diseases. However, clinical antibiotic therapy for *H. pylori* is limited by continuously decreased therapeutic efficacy and side effects to symbiotic bacteria. Herein, we develop an in vivo activatable pH-responsive graphitic nanozyme, PtCo@Graphene (PtCo@G), for selective treatment of *H. pylori*. Such nanozymes can resist gastric acid corrosion, exhibit oxidase-like activity to stably generate reactive oxygen species only in acidic gastric milieu and demonstrate superior selective bactericidal property. C_18_-PEG_n_-Benzeneboronic acid molecules are modified on PtCo@G, improving its targeting capability. Under acidic gastric pH, graphitic nanozymes show notable bactericidal activity toward *H. pylori*, while no bacterial killing is observed under intestinal conditions. In mouse model, high antibacterial capability toward *H. pylori* and negligible side effects toward normal tissues and symbiotic bacteria are achieved. Graphitic nanozyme displays the desired enzyme-like activities at corresponding physiological sites and may address critical issues in clinical treatment of *H. pylori* infections.

## Abstract (web summary)

*Helicobacter pylori* is a major cause of gastric diseases, but the standard therapy is limited by continuously decreased therapeutic efficacy and side effects to symbiotic bacteria. Here, the authors develop a pH-responsive graphitic nanozyme that is active under low pH gastric conditions, but inactive in intestines, for selective treatment of *H. pylori* infections.

## Introduction

*H. pylori* is a gram-negative microaerobic spiral-shaped bacterium that mainly colonizes in the human stomach and is a major etiological factor in many gastric diseases, such as gastritis, gastric ulcers, and gastric cancer^1–5^. It is reported that around 50% of the global population has been infected by *H. pylori*^6,7^. Triple therapy, as the standard first-line therapy in the clinical treatment of *H. pylori* infection, involves a proton pump inhibitor (omeprazole) and two antibiotics (amoxicillin, clarithromycin/metronidazole)^8,9^. However, the insufficient retention time of antibiotics in gastric lumen^10^ and the degradation of antibiotics by gastric acid^11^ severely affect the antimicrobial activity of antibiotics. Furthermore, owing to the constant emergence of drug-resistant bacteria, the therapeutic efficacy of triple therapy is also decreased^12–14^. In addition, administration of high-dose antibiotics may cause severe side effects for symbiotic bacteria which are closely related to many otherwise beneficial physiological and metabolic processes^15,16^. Gut microflora dysbiosis is known to result in many diseases. Therefore, it is imperative to develop an alternative therapeutic strategy to selectively treat *H. pylori* with superior antibacterial efficacy, while also maintaining a healthy balance for the commensal bacteria.

Since ferromagnetic nanoparticles were unexpectedly discovered with peroxidase-like activity in 2007^17^, hundreds of nanomaterials have been reported to have enzyme-like activities. Unlike the prohibitively high-cost and unstable properties of natural enzymes, nanozymes have superior advantages, such as low-cost, high stability, easy large-scale production, and multiple functionalities^18–20^. In addition, the superior peroxidase- and oxidase-like properties of nanozymes can mimic the functions of natural enzymes to catalyze the generation of highly toxic reactive oxygen species (ROS)^21^. Because of such features, nanozymes, as a new generation of antibacterial agents, have been widely used in the fields of antibacterial applications^22^. Versatile nanozymes, such as metal nanomaterials^23,24^, metal oxide/sulfide or their nanocomposites^25–29^, and carbon-based nanomaterials^30^ have been reported to combat bacteria. Most nanozymes are mainly used for killing bacteria under physiological conditions, such as wound infections. However, to the best of our knowledge, nanozymes for the treatment of bacteria in a specific environment, such as *H. pylori* in the stomach, have been rarely reported. Under strong acidic conditions (pH 0.9–1.5) of gastric juice^31^, nanozymes must resist the corrosion of gastric acid and still possess high enzyme-like activity. Furthermore, for in vivo applications, the present problem of nanozymes is how to control them to show desired enzyme-like activity only in the corresponding physiological sites^32^. This site-directed activation should improve the therapeutic efficacy of nanozymes and reduce harmful effects against commensal bacteria and sites not infected. Consequently, developing versatile nanozyme materials is urgently needed.

Here, we fabricate a superstable graphitic nanozyme with pH-responsive oxidase-like activities and selective antibacterial capability toward *H. pylori* infection. Such nanozymes are made of a graphene-isolated PtCo nanocrystal functionalized with the bacteria-binding molecule C_18_-PEG_n_-Benzeneboronic acid (PtCo@G@CPB). The graphene grown on the surface of PtCo alloys protects the properties of the metal core from interference with the external environment, especially under harsh conditions. Under gastric acid conditions, the oxidase-like activity of PtCo@G can be activated with superior stability to catalyze the generation of ROS for antibacterial applications. However, under intestinal neutral conditions, the oxidase-like activity of the PtCo@G will be suppressed, demonstrating minimal toxicity. In the *H. pylori*-infected mouse model, PtCo@G@CPB shows high antibacterial activity toward *H. pylori* and low side effects toward normal tissues and symbiotic bacteria. Furthermore, the intrinsic magnetic resonance imaging (MRI, from the Co element) and Raman imaging (from the graphitic shell) properties of PtCo@G@CPB can be used to monitor the distribution of nanomaterials in vivo without introducing additional marker molecules. Overall, this stable PtCo@G@CPB with in vivo activated oxidase-like activity show promise to address the critical challenges that exist in the clinical treatment of *H. pylori* infections.

## Results and Discussion

### Synthesis and characterization of graphitic nanozyme

**Fig. 1:**
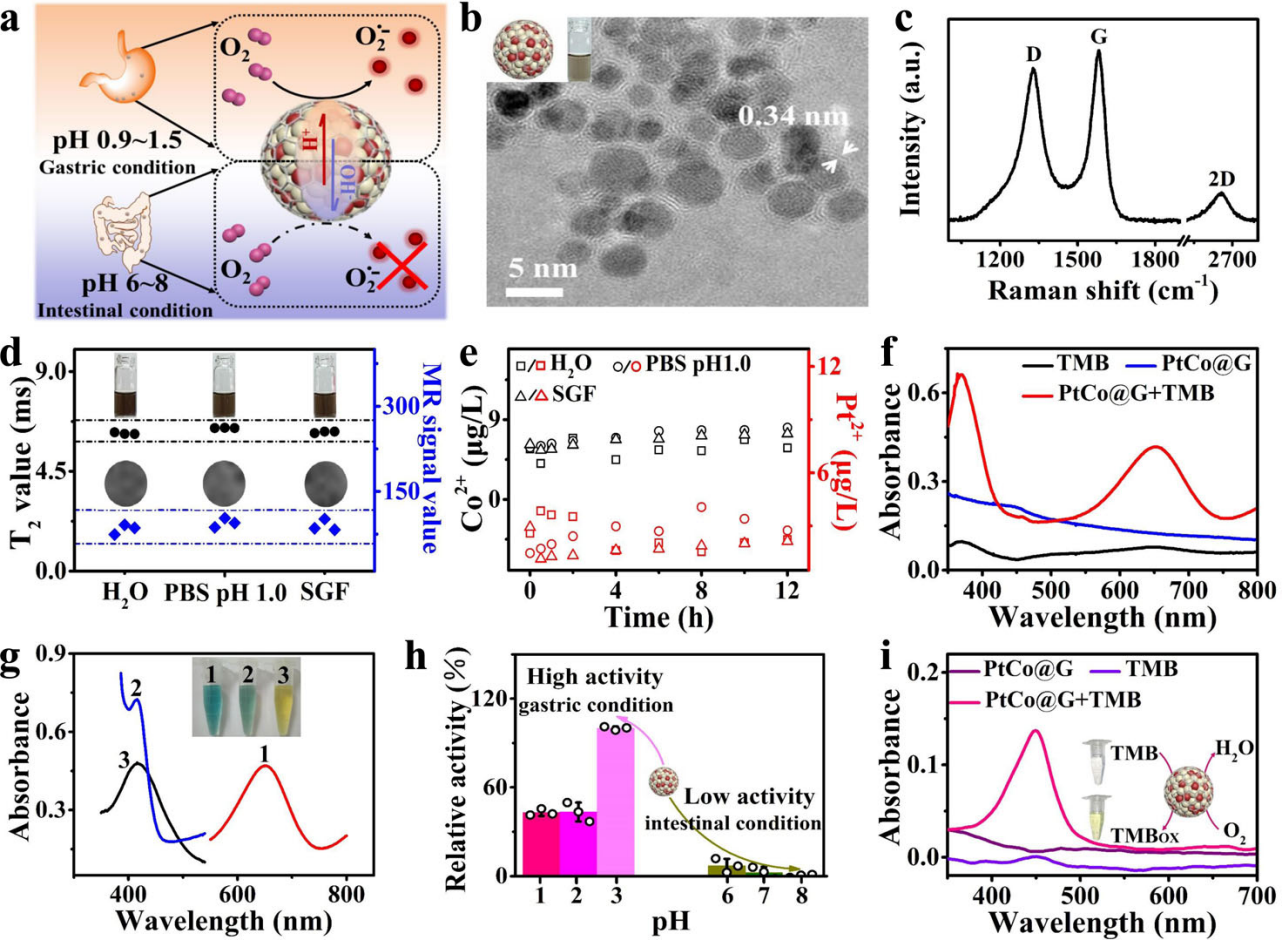
Characterization of pH-responsive graphitic nanozyme. **a** Schematic illustrating selective activated pH-responsive oxidase-like activity of PtCo@G in gastric acid conditions. **b** TEM image of PtCo@G. Insert shows corresponding structure diagram of PtCo@G (including the PtCo nanocrystal core and graphitic shell) and digital photos of the PtCo@G solution. **c** Raman spectrum of PtCo@G. **d** The digital photos, MR T_2_ relaxivity, T_2_-weighted phantom images and corresponding quantitative data analysis on T_2_-weighted phantom images of PtCo@G in H_2_O, PBS (pH 1.0), and SGF after 12 h incubation. **e** Profiles for Co^2+^ and Pt^2+^ release from PtCo@G nanocrystals in H_2_O, PBS (pH 1.0), and SGF. **f** UV-Vis absorbance spectra of TMB in different reaction systems: TMB, PtCo@G, and PtCo@G + TMB in a pH 4.0 PBS buffer after 30 min incubation. **g** PtCo@G catalyzes the oxidation of various substrates to produce different color reactions. (1) TMB, (2) ABTS, and (3) OPD. Insert images show corresponding visual color changes. **h** Selective activated pH-responsive oxidase-like activity of PtCo@G under gastric acid conditions. **i** Oxidase-like activity of the PtCo@G in SGF. Insert images show corresponding visual color changes and the reaction between TMB and PtCo@G in SGF. The data indicate the means and SD from three parallel experiments. Source data are provided as a Source Data file.

We rationally designed graphene-isolated PtCo (PtCo@G) nanozymes. The synthesis of PtCo@G was carried out by a simple one-pot method. Core-shell PtCo@G consists of a PtCo nanocrystal core encapsulated in a graphitic shell. The Pt element of the metal core endowed PtCo@G with nanozyme activity. The Co element and the graphitic shell with unique Raman vibration bands enabled the nanozyme to perform multimodal MRI and Raman imaging. The graphitic shell of PtCo@G also afforded the nanozyme with superior stability. As shown in Fig. 1a, under gastric acid conditions, PtCo@G exhibited an intrinsic oxidase-like property to catalyze superoxide radical (O_2_^•−^) generation, as one of the highly toxic ROS, to attack the bacterial membrane and cause cytoplasmic leakage, resulting in bacterial death^24^. On the other hand, under neutral intestinal conditions, the oxidase-like activity of PtCo@G would be suppressed and demonstrate negligible toxicity. Such PtCo@G could have in vivo activatable pH-responsive nanozyme activities. Transmission electron microscopy (TEM, Supplementary Fig. 1) and high-resolution TEM (HRTEM, Fig. 1b) images of the samples indicated that the synthesized PtCo@G displayed a core-shell structure with an average diameter of 3 nm (structure diagram of PtCo@G, insert of Fig. 1b). The space between the outer shell layers of the PtCo@G was ~0.34 nm, consistent with the interlayer distance of graphite, suggesting that the PtCo nanocrystal core was encapsulated by few-layer graphene. Insert from Fig. 1b displayed the digital photos of the PtCo@G solution. Ultraviolet-visible spectra of the PtCo@G solution indicated that the absorbance band located at ~248 nm originated from the graphitic shell (Supplementary Fig. 2). The hydrodynamic diameter of PtCo@G was ~18 nm (Supplementary Fig. 3a). PtCo@G exhibited negative charge after treatment with concentrated HCl solution to remove poorly encapsulated PtCo@G (ζ = −20 mV, Supplementary Fig. 3b). In Raman spectroscopy, PtCo@G revealed three prominent Raman vibration bands, around 1340, 1600, and 2670 cm^−1^, corresponding to disordered (D), graphitic (G), and intervalley double-resonance Raman band (2D) vibrational modes of graphitic shells, respectively (Fig. 1c).

The stability of PtCo@G in gastric acid conditions would directly influence its nanozyme activity. As a real gastric acid environment mimic, simulated gastric fluid (SGF), including pepsin, sodium chloride, and hydrochloric acid^33^, was prepared according to United States Pharmacopoeia 24. Various characterization methods were utilized to monitor the stability of PtCo@G in SGF. Fig. 1d showed the digital photos, MR T_2_ relaxivity and T_2_-weighted phantom images of PtCo@G in H_2_O, phosphate buffer solution (PBS, pH 1.0) and SGF after 12 h incubation, and almost no difference was observed. Moreover, the hydrodynamic diameter of PtCo@G was detected and showed almost no difference, indicating that PtCo@G exhibited excellent stability in SGF (Supplementary Fig. 4). To further demonstrate the stability of PtCo@G nanozyme in SGF, dissolution experiments were carried out, and the results showed that no obvious Co^2+^ and Pt^2+^ were released from the PtCo@G nanocrystals (Fig. 1e and Supplementary Fig. 5), demonstrating the robust stability of PtCo@G in SGF.

The oxidase-like properties of PtCo@G were then investigated while utilizing 3,3′,5,5′-tetramethylbenzidine (TMB) as the molecular probe. The oxidation product of TMB could form charge transfer complex with TMB and display a characteristic absorbance at 652 nm (Fig. 1f)^34^. The oxidase-like activity of PtCo@G was further characterized with several different oxidase substrates, such as 2,2′-azino-bis(3-ethylbenzthiazoline-6-sulfonic acid) (ABTS) and o-phenylenediamine (OPD). Experimental results demonstrated that PtCo@G could catalyze the fast oxidation of both ABTS and OPD with the appearance of the characteristic color and corresponding UV-vis spectra (Fig. 1g). Meanwhile, the catalytic activity of PtCo@G was further systematically investigated at different time increments, solution pH and temperatures. As shown in Supplementary Fig. 6, PtCo@G could clearly catalyze TMB oxidation, as evidenced by the time-dependent increase in the maximum absorbance. Interestingly, PtCo@G exhibited excellent catalytic activity over an extensive pH and temperature range (Supplementary Fig. 7). The oxidase-like activity of the PtCo@G nanozyme was pH-dependent and could only be activated under acidic conditions. Even at strong acidic pH (pH 1–2) levels, it was noteworthy that the as-prepared PtCo@G also exhibited an intrinsic oxidase-like property, about 43% at pH 1.0 compared to that at pH 3.0, which would facilitate its bio-applications at special physiological conditions, such as the stomach. The mechanism of the pH-dependent oxidase-like activity of PtCo@G may origin from the different influences of solution pH on the dissociative adsorption of O_2_ on the PtCo@G surfaces^35,36^. Besides its intrinsic oxidase-like property, the graphitic nanozyme could act simultaneously with robust and effective peroxidase-like activity. We investigated the peroxidase-like activity of PtCo@G by following the catalysis of the peroxidase substrates TMB, ABTS, and OPD by H_2_O_2_ (Supplementary Fig. 8a, b, c). Like other nanomaterial-based peroxidase mimics, the peroxidase-like activity of PtCo@G was also dependent on pH (Supplementary Fig. 9a) and temperature (Supplementary Fig. 9b). All of the above experimental results demonstrated that PtCo@G exhibited intrinsic oxidase/peroxidase-like activity, even at strong acidic pH conditions (pH 1–2). We investigated the possible mechanism of the oxidase/peroxidase-like activity of PtCo@G at strong acidic pH. To monitor the possible active intermediate in the reaction system, various colorimetric and fluorescence probes were used to evaluate the ability of PtCo@G to generate ROS. At strong acidic pH condition (PBS, pH 1.0), TMB could be oxidized by PtCo@G and produced a yellow color (Supplementary Fig. 10a). The catalytic reaction was shown as Supplementary Fig. 10b^37^. Hydroethidine (HE), a highly selective fluorescence probe for superoxide radical (O_2_^•−^), could be oxidized by O_2_^•−^ to generate the fluorescent product ethidium (E^+^). Upon adding PtCo@G, an obvious increase in fluorescence intensity of HE occurred, indicating that PtCo@G possessed the exceptional capability to generate O_2_^•−^ at strong acidic pH (Supplementary Fig. 11a, b, c). The peroxidase-like property of PtCo@G could decompose H_2_O_2_ species to generate hydroxyl radical (•OH). Here, we used terephthalic acid (TA) and 5-(Diethoxyphosphoryl)-5-methyl-1-pyrroline-N-oxide (DEPMPO) to track the formation of •OH in the reaction system (Supplementary Figs. 12 and 13). Experimental results indicated that the •OH species were mainly generated from the decomposition of H_2_O_2_ catalyzed by PtCo@G.

We then compared the oxidase-like activity of PtCo@G in gastric acid conditions and neutral intestinal conditions. As shown in Fig. 1h, in the gastric condition, PtCo@G nanozyme exhibited excellent oxidase-like activity. However, PtCo@G showed almost no oxidase-like activity in the neutral intestinal condition which indicated the site selectivity of such nanozyme. The oxidase-like property of PtCo@G in SGF was further demonstrated by using TMB as oxidase substrate. Similar to PBS (pH 1.0), TMB was oxidized quickly by PtCo@G and generated obvious yellow color, which indicated that PtCo@G exhibited a distinct oxidase-like property in SGF (Fig. 1i).

### pH-responsive PtCo@G@CPB for selective treatment of H. pylori in vitro

**Fig. 2:**
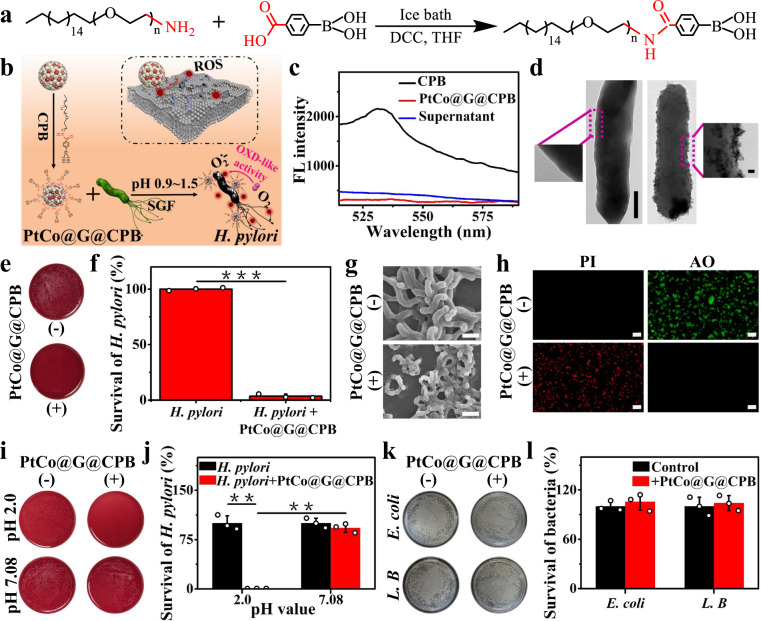
C_18_-PEG_n_-benzeneboronic acid (CPB) synthesis and PtCo@G@CPB selective treatment of *H. pylori* in vitro. **a** Schematic illustration of C_18_-PEG_n_-Benzeneboronic acid synthesis. **b** Schematic of CPB modified PtCo@G nanocrystals and antibacterial mechanism of PtCo@G@CPB against *H. pylori*. **c** Fluorescence spectra characterization of CPB functionalized PtCo@G. **d** TEM images showing the ability of PtCo@G@CPB to target *H. pylori. H. pylori* incubated with PtCo@G (left) and PtCo@G@CPB (right) at SGF for 0.5 h. Scale bar: left 0.5 μm, right 20 nm. **e** Photographs of *H. pylori* colonies. **f** The antibacterial activity of PtCo@G@CPB against *H. pylori*. *P* values were calculated by the Student’s two-sided *t*-test: ***p < 0.001. **g** SEM images of *H. pylori* treatment without or with PtCo@G@CPB. Scale bar: 1.0 μm. **h** Fluorescence images of live (green) and dead (red) *H. pylori* after incubation without or with PtCo@G@CPB. PI: propidium iodide, AO: acridine orange. Scale bar: 10 μm. **i** Photographs of *H. pylori* colonies under various conditions. **j** Relative bactericidal activity of PtCo@G@CPB against *H. pylori* under gastric acid environment and neutral intestinal environment. *P* values were calculated by the Student’s two-sided *t*-test: **p < 0.01. **k** Photographs of *E. coli* and *L. B* colonies under different conditions. **l** The relative antibacterial activity of PtCo@G@CPB against intestinal symbiotic bacteria. The concentration of PtCo@G@CPB used in the above antibacterial experiments was 70 μg/mL. The data indicate the means and SD from three parallel experiments. Source data are provided as a Source Data file.

With unique enzyme-like properties, we then investigated the antibacterial application of PtCo@G nanozymes in vitro. The effective target *H. pylori* capability of PtCo@G could significantly enhance antibacterial efficacy by increasing the concentration of ROS around the bacterial surface and reduce the damage to normal tissues^38,39^. Boronic acid is a bacteria-binding molecule, which can reversibly bind peptidoglycan from the surface of the bacterial cell wall, leading to the specific capture of *H. pylori*^40–42^. Therefore, to achieve selective adherence to *H. pylori* and a high local concentration of ROS around the bacterial membrane, we synthesized C_18_-PEG_n_-Benzeneboronic acid through the chemical bonding between carboxylic groups of 4-carboxyphenylboronic acid and amine groups of C_18_-PEG_n_-NH_2_ (Fig. 2a) and functionalized the molecule on the surface of PtCo@G to target *H. pylori*. The synthesized CPB molecules were characterized by ^1^H-NMR (Supplementary Fig. 14). The peak of 7.79 ppm in ^1^H-NMR demonstrated the generation of an amide bond, indicating the successful synthesis of CPB molecules. As shown in Fig. 2b, the synthesized CPB molecule has a hydrophobic alkyl tail, making it easy to functionalize on the surface of PtCo@G through hydrophobic interactions, resulting in specific targeting of *H. pylori*. PtCo@G@CPB on the *H. pylori* could catalyze ROS generation, leading to high local ROS concentration around the bacterial membrane. The ROS on the surface of *H. pylori* cell wall could directly disrupt the bacterial membranes and cause cytoplasmic leakage, eventually resulting in bacterial death^43,44^. The functionalization of CPB molecule on the surface of PtCo@G was characterized by fluorescence and Raman spectroscopy (Fig. 2c and Supplementary Fig. 15). The fluorescence of fluorophore could be quenched by the graphitic shell of PtCo@G through the fluorescence resonance energy transfer effect. After interacting with PtCo@G, the fluorescence of the boronic acid group of CPB significantly decreased, indicating the successful functionalization of PtCo@G with CPB molecules. Furthermore, PtCo@G@CPB exhibited Raman vibration band around 3150 cm^−1^, corresponding to OH group vibrational mode of boronic acid group on CPB molecules, which also indicated successful modification of CPB on the surface of PtCo@G. The oxidase-like property of PtCo@G@CPB was also investigated. As shown in Supplementary Fig. 16, the modification of CPB scarcely affected the oxidase-like property of PtCo@G in SGF solution. Under the microaerobic conditions, PtCo@G@CPB also exhibited excellent oxidase-like activity (Supplementary Fig. 17). The PtCo@G@CPB showed Michaelis-Menten kinetics in TMB colorimetric reaction. The experimental results also demonstrated that the enzyme-like properties of PtCo@G@CPB were pH-dependent and possessed intrinsic oxidase-like property even under microaerobic atmosphere as indicated by the *K*_m_ and *V*_max_ values (Supplementary Fig. 18 and Supplementary Tables 1 and 2). Then, PtCo@G@CPB was examined for its targeting capability with *H. pylori*. Upon incubating *H. pylori* with PtCo@G@CPB, we found numerous PtCo@G@CPB attached on the surface of *H. pylori*, indicating superior bacteria-targeting ability (Fig. 2d).

The antibacterial capability of PtCo@G@CPB with different concentrations against *H. pylori* was then assessed in SGF solution. The PtCo@G@CPB significantly inhibited the survival of *H. pylori* in a dose-dependent manner (Supplementary Fig. 19). Through conventional plate colony counting experiments and relative bacteria activity tests (Supplementary Fig. 20 and Fig. 2e, f), PtCo@G@CPB showed significant bactericidal capabilities. Moreover, scanning electron microscopy (SEM) characterization was utilized to analyze the morphology of bacteria under various conditions. As shown in Fig. 2g and Supplementary Fig. 21, *H. pylori*, without being incubated with PtCo@G@CPB, exhibited typically spiral rod-shaped morphology with intact cell walls, while they completely lost their cellular integrity after being treated with PtCo@G@CPB. To further examine the bactericidal property of PtCo@G@CPB, fluorescence-based live-dead bacterial viability assays were performed (Fig. 2h). Without PtCo@G@CPB incubation, most bacteria remained alive. Remarkably, after incubation with PtCo@G@CPB, the number of dead bacteria dramatically increased. Then, we compared the bactericidal activity of PtCo@G@CPB against *H. pylori* under gastric acid environment to that under neutral intestinal environment (Fig. 2i, j). Upon incubation of *H. pylori* with PtCo@G@CPB at pH 2.0 (acidic gastric condition), notable bactericidal activity was noted, while no bacterial killing was observed at pH 7.0 (neutral intestinal condition). The cytotoxicity of PtCo@G@CPB was investigated through CCK-8 assay. Negligible inhibition of proliferation was observed in GES-1 cells stained with PtCo@G@CPB (Supplementary Fig. 22). On the other hand, *Escherichia coli* (*E. coli*) and *Lactobacillus* (*L. B*), important physiological flora in human or animal intestines, were used to investigate the bactericidal activity of PtCo@G@CPB under relatively neutral intestinal conditions. As shown in Fig. 2k, l, PtCo@G@CPB showed very low antibacterial activity against *E. coli* and *L. B*, indicating that PtCo@G@CPB does not kill symbiotic intestinal bacteria under relatively neutral conditions with superior biocompatibility. In a word, all of the above experimental results indicate that PtCo@G@CPB is able to selectively kill *H. pylori* under gastric acid conditions via its pH-responsive oxidase-like activity.

### In vivo activation of PtCo@G@CPB for selective treatment of *H. pylori*

**Fig. 3:**
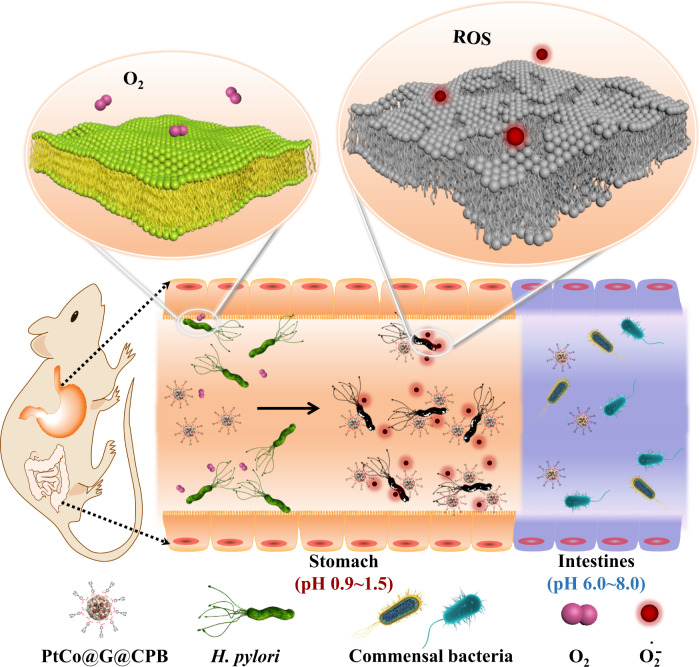
The schematic of selective sterilization of *H. pylori* in vivo based on PtCo@G@CPB nanozyme. In the acidic stomach condition, the PtCo@G@CPB nanozyme could specifically target *H. pylori* and catalyze ROS formation, showing significantly enhanced antibacterial efficacy. After entering neutral intestinal condition, the oxidase-like activity of PtCo@G@CPB would be suppressed, and it showed minimal toxicity towards commensal bacteria.

The in vivo bactericidal capabilities of PtCo@G@CPB were then investigated. As illustrated in Fig. 3, under an acidic gastric condition, PtCo@G@CPB could catalyze ROS formation, resulting in high local ROS concentration around the bacterial membrane and showing excellent antibacterial efficiency. However, after entering neutral intestinal conditions, the oxidase-like activity of PtCo@G@CPB would be suppressed, allowing for its biocompatibility with symbiotic intestinal bacteria.

**Fig. 4:**
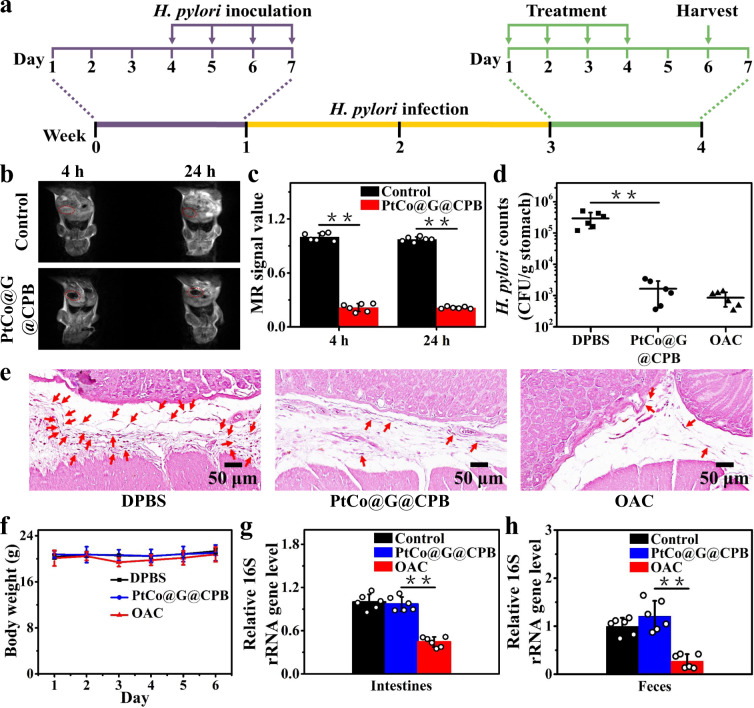
In vivo therapeutic effect of PtCo@G@CPB. **a** The study protocol of *H. pylori* inoculation, infection development, and treatment in BALB/c mice. **b** Retention time of PtCo@G@CPB in the stomach of *H. pylori*-infected mouse. **c** Corresponding data analysis of mouse stomach in **b**. Data are presented as mean ± SD (*n* = 6 biologically independent mice). *P* values were calculated by the Student’s two-sided *t*-test: **p < 0.01. **d** Bacterial burden in the stomach of *H. pylori*-infected mice treated with DPBS, PtCo@G@CPB, and triple therapy. Data are presented as mean ± SD (*n* = 6 biologically independent mice). *P* values were calculated by the Student’s two-sided *t*-test: **p < 0.01. **e** Gram staining of slice from the gastric mucosa receiving DPBS, PtCo@G@CPB, and OAC treatments. Red arrows point to bacteria. **f** Bodyweight change of mice following treatment of various formulations as in **d**. Data are presented as mean ± SD (*n* = 6 biologically independent mice). The side effects of PtCo@G@CPB against symbiotic bacteria in **g** intestine and **h** feces of mice. Data are presented as mean ± SD (*n* = 6 biologically independent mice). *P* values were calculated by the Student’s two-sided *t*-test: **p < 0.01. Source data are provided as a Source Data file.

We evaluated the therapeutic efficacy of PtCo@G@CPB nanozymes against *H. pylori* infection in vivo. Prior to the therapy, the *H. pylori* infection model was established using BALB/c mice. As shown in Fig. 4a, each mouse was inoculated with 1.5 × 10^7^ CFU *H. pylori* by oral gavage once daily for 4 days. After 2 weeks of inoculation, the quantification of bacteria burden in the mouse stomach was 1.8 × 10^5^ CFU/g of stomach tissue (Supplementary Fig. 23a, b). Furthermore, the Gram staining of *H. pylori*-infected mouse gastric mucosa also demonstrated the presence of bacteria in stomach tissue (Supplementary Fig. 23c). This indicated that the *H. pylori*-infected mouse model was established successfully. Next, the retention time of PtCo@G@CPB in the stomach of *H. pylori*-infected mouse was investigated. The MR imaging of PtCo@G@CPB was used to study its retention in the stomach. At 4 h after intragastric administration, the T_2_-weighted phantom imaging of PtCo@G@CPB in *H. pylori*-infected mouse stomach was clear, and the distinct imaging result was almost unchanged until 24 h (Fig. 4b, c), which was relatively long compared with the reported gastric emptying times of mice^45^. To further identify the retention of PtCo@G@CPB in the stomach of the *H. pylori*-infected mouse, the whole mouse stomach was excised at 24 h after intragastric administration, and the D- and G-bands of the PtCo@G were utilized for Raman imaging of gastric tissue (Supplementary Fig. 24). The distinct Raman D and G peaks of the PtCo@G could be easily identified in the mouse gastric tissue treated with PtCo@G@CPB. In addition, the retention of PtCo@G@CPB in the stomach of mouse was also certified by determining the amounts of Pt in the stomach organs of mice at 24 h after intragastric administration (Supplementary Fig. 25). The apparent presence of the PtCo@G@CPB indicated the effective retention in the stomach of *H. pylori*-infected mouse.

After 2 weeks of inoculation, the *H. pylori*-infected mice were divided into three groups (*n* = 6) and intragastrically administered with DPBS, PtCo@G@CPB and triple therapy OAC (omeprazole, amoxicillin, and clarithromycin) once a day for 4 days, respectively. On each day of treatment, mice received proton pump inhibitor (omeprazole) 30 min before intragastric administration of antibiotic to neutralize gastric acid and prevent potential antibiotic degradation. Mice treated with PtCo@G@CPB showed bactericidal efficacy in the stomach similar to that of triple therapy OAC, with bacterial burden decreasing in the stomach 100-fold over that of the control group (treated with DPBS) (Fig. 4d). Gram staining of *H. pylori*-infected mice gastric tissue treated with DPBS, PtCo@G@CPB, and OAC, respectively, was also investigated (Fig. 4e). Compared with the control group, the Gram staining of gastric tissue treated with PtCo@G@CPB indicated no obvious bacterial presence in the stomach, similar to that of the OAC group. All these results indicated that the PtCo@G@CPB nanozymes were effectively activated in the stomach and demonstrated superior *H. pylori* killing capability in vivo.

The in vivo toxicity of PtCo@G@CPB was also explored during the treatments. No obvious change in the mice’s body weight was observed during the PtCo@G@CPB treatment, which indicated the low toxicity of PtCo@G@CPB nanozymes (Fig. 4f). To further analyze the toxicity of PtCo@G@CPB toward mice stomach, at the sixth day of gavage, all mice were euthanized, and the gastric tissue obtained from the mice was stained with hematoxylin and eosin. The gastric tissue of mice treated with PtCo@G@CPB showed no obvious inflammation or injury with a clear layer of epithelial cells. This was similar to the gastric sample of mice treated with DPBS (Supplementary Fig. 26a, b, c). PtCo@G@CPB toxicity was further evaluated by examining its distribution in different organs of the mice after treatment (Supplementary Fig. 27). Almost no PtCo@G@CPB was observed in the visceral organs, which also demonstrated that oral administration with PtCo@G@CPB had superior biocompatibility. More importantly, the effects of PtCo@G@CPB on the symbiotic bacteria were also studied. PtCo@G@CPB showed no significant killing of symbiotic bacteria in the intestine and feces of mice, while triple-therapy OAC treatment killed symbiotic bacteria in the intestine and feces by 56% and 73%, respectively (Fig. 4g, h). All experimental results indicated the low side effects of PtCo@G@CPB toward normal tissues and intestinal symbiotic bacteria. The pH-responsive graphitic nanozymes could only be activated in the stomach and demonstrated selective bactericidal capabilities in vivo.

In conclusion, we developed a superstable in vivo activatable pH-responsive graphitic nanozyme for selective treatment of *H. pylori* infection. Under the gastric acid environment, the oxidase-like activity of PtCo@G@CPB nanozyme was effectively activated and could specifically target *H. pylori*, leading to significantly enhanced antibacterial efficacy by increasing the local concentration of ROS around the bacterial surface. Such in vivo activated nanozyme showed bactericidal capabilities comparable to the standard first-line triple therapy. However, the oxidase-like property of PtCo@G@CPB was suppressed in the neutral intestinal condition, and it demonstrated minimal toxicity towards symbiotic bacteria. Overall, such gastric juice-activated nanozyme activity of PtCo@G@CPB shows promise for overcoming the critical challenges in selective treatment of *H. pylori* infection.

## Methods

### Materials

Fumed silica was purchased from Aladdin Industrial Corporation (Shanghai, China). Chloroplatinic acid (H_2_PtCl_6_·6H_2_O, 99.9%) and Co(NO_3_)_2_·6H_2_O were purchased from Changsha Chemical Reagents Company (Changsha, China). Gastric pepsin, TMB, horseradish peroxidase, ABTS, OPD, TA, and HE were purchased from Sigma-Aldrich China (Shanghai, China). DEPMPO was purchased from Santa Cruz Biotechnology China (Shanghai, China). *Helicobacter pylori* (ATCC 43504) was purchased from American type culture collection (ATCC). BALB/c female mice (3–4 weeks old) were obtained from the Hunan SJA Laboratory Animal Co., Ltd (Changsha, China) and all animals were carried out in accordance with the Guide for the Care and Use of Laboratory Animals and used under protocols approved by the Institutional Animal Care and Use Committee of Hunan University. The GES-1 cell line was obtained from iCell Bioscience Inc. (Shanghai, China). All other chemicals of analytical reagent grade were obtained from Changsha Chemical Reagents Company (Changsha, China) and used as received without further purification. Ultrapure water (resistance > 18.2 MΩ cm^−1^) was used throughout all experiments.

### Characterization and instruments

All Raman measurements were performed in a Renishaw’s InVia Raman system with 633 nm laser excitation (Renishaw, UK). Ultraviolet-visible spectra were recorded by a UV-2450 UV-vis spectrophotometer (Shimadzu, Japan). Hydrodynamic diameters and Zeta potentials were measured by a DLS system (Malvern, UK). TEM images were taken with a JEM-2010 (JEOL, Japan). SEM images were measured by JSM-6700F (Japan). Fluorescence spectra were performed by HORIBA Fluoromax-4 (HORIBA, USA). Electron paramagnetic resonance (EPR) measurements were recorded by JES-FA200 electron spin resonance (JEOL, Japan). A Minispec MQ60 60 MHz TD NMR broadband spectrometer (Bruker, Germany) was used to measure T_2_ relaxivity. T_2_-weighted MR imaging was taken by the 1.5 T small animal MR scanner (MRI 1.5 T; Shining Global Science and Education Equipment Co., Ltd., Shanghai, China). Inductively coupled plasma mass spectrometry (ICP-MS, Agilent, USA) was utilized to measure the concentrations of released Co^2+^ and Pt^2+^ in the solution.

### Preparation and characterization of PtCo@G nanocrystals

Magnetic graphitic PtCo nanocrystals (PtCo@G) were prepared through a chemical vapor deposition (CVD) method. First, fumed silica (1.00 g) was impregnated with chloroplatinic acid (5.0 mL, 1.0%) and Co(NO_3_)_2_·6H_2_O (0.073 g) in methanol and sonicated for 1 h. Methanol was removed, and the mixture was dried at 45 °C. Then, the powder was placed into a methane CVD chamber for growth with a methane flow of 150 s.c.c.m. for 5 min at 1000 °C. After growth, hydrofluoric acid was used to etch the silica, followed by collecting the PtCo@G with a magnet. To achieve water-soluble PtCo@G nanomaterials, 100 μL saturated octadecyl-polyethylene glycol (C_18_-PEG) solution was added into the PtCo@G solution and ultrasonicated for 1.5 h. Then, the excess C_18_-PEG was washed off and the product was stored at 4 °C for future use. TEM (JEOL, Japan) was used to characterize the morphology of PtCo@G.

### Comparison of stability of PtCo@G in H_2_O, PBS (pH 1.0), and SGF

Equal amounts of PtCo@G were added into H_2_O, PBS (pH 1.0), and SGF and incubated for 12 h. Then the digital photos, hydrodynamic diameter, MR T_2_ relaxivity and T_2_-weighted phantom images of PtCo@G in H_2_O, PBS (pH 1.0), and SGF were measured by DLS (Malvern, UK), Minispec MQ60 60 MHz TD NMR broadband spectrometer (Bruker, Germany) and 1.5 T small animal MR scanner (MRI 1.5 T; Shining Global Science and Education Equipment Co., Ltd., Shanghai, China), respectively.

### Dissolution experiments

To detect the release of Co^2+^ and Pt^2+^ from PtCo@G nanocrystals, dissolution experiments were carried out. Equal amounts of PtCo@G were added into H_2_O, PBS (pH 1.0), and SGF and incubated for different times (0, 0.5, 1, 2, 4, 6, 8, 10, 12, 16, 20, and 24 h) with constant shaking. The PtCo@G nanocrystals were removed from the solution by centrifugation (15,294 × *g*, 30 min), and the supernatant was stabilized to 5.0 mL. The concentrations of released Co^2+^ and Pt^2+^ in the solution were measured with inductively coupled plasma mass spectrometry (ICP-MS, Agilent, USA).

### Oxidase-like property of PtCo@G

The oxidation of TMB by PtCo@G in phosphate buffer produced a blue color with major absorbance peaks at 652 nm. In a typical test, certain amounts of TMB (final concentration 500 μM) and PtCo@G were added into buffer solution (25 mM PBS, pH 4.0), and then UV-Vis absorption spectra were recorded at different times using a UV-2450 UV-vis spectrophotometer (Shimadzu, Japan). Other organic dyes, such as ABTS and OPD, were also used to further confirm the oxidase-like property of PtCo@G in the same way.

### Peroxidase-like property of PtCo@G

The oxidation of TMB by PtCo@G/H_2_O_2_ in 25 mM phosphate buffer (pH 4.0) produces a blue color with major peaks at 652 nm. Certain amounts of TMB (final concentration 500 μM), H_2_O_2_ (final concentration 100 μM), and PtCo@G were added into buffer solution, and then UV-Vis absorption spectra were recorded at different times using a UV-2450 UV-vis spectrophotometer (Shimadzu, Japan). Other organic dyes, such as ABTS and OPD, were also used to further confirm the peroxidase-like property of PtCo@G.

### Oxidase-like and peroxidase-like property of PtCo@G in PBS (pH 1.0)

For assay of the product O_2_^•−^, hydroethidine (HE) was used as a probe, which could be easily oxidized by O_2_^•−^ to generate the highly fluorescent product ethidium (E^+^). In a typical procedure, HE was added into the reaction solution (PBS, pH 1.0) for 30 min, and then the fluorescence spectra of the samples were collected using fluorescence spectroscopy (HORIBA Fluoromax-4, USA).

For assay of the product •OH, TA, and DEPMPO, which were used as probes, could easily react with •OH to form a highly fluorescent product (TAOH) and EPR spin adduct (DEPMPO-OH). In experimental procedure, TA and DEPMPO were added into reaction solutions (PBS, pH 1.0). Then, the fluorescence spectra and EPR of the samples were performed by fluorescence spectroscopy (HORIBA Fluoromax-4, USA) and EPR spectroscopy (JEOL, Japan).

### Oxidase-like property of PtCo@G in simulated gastric fluid (SGF)

Chemicals were added into SGF in an order of certain amounts of PtCo@G and TMB (final concentration 500 μM). The oxidation of TMB by PtCo@G in SGF produces a yellow color, and UV-Vis absorption spectra were recorded using a UV-2450 UV-vis spectrophotometer (Shimadzu, Japan).

### The kinetic assays for oxidase-like and peroxidase-like activities of PtCo@G@CPB

The kinetic measurements for oxidase-like activity of PtCo@G@CPB with TMB as the substrate were performed by adding different amounts (1, 2, 4, 6, 8, and 10 μL) of TMB solutions (10 mM) into reaction solution (final volume was 200 μL). The reaction system was performed under different pH solutions or different oxygenated environments, respectively. The kinetic assays for peroxidase-like activity of PtCo@G@CPB with H_2_O_2_ as the substrate were performed by adding certain amount of TMB solution (final concentration was 0.5 mM) and different amounts (1, 2, 4, 6, 8, 10 μL) of H_2_O_2_ solutions (100 mM) into reaction system (final volume was 210 μL) with different pH values. The Michaelis-Menten constant (*K*_m_) was calculated according to the Lineweaver-Burk Plot: 1/v = *K*_m_/(*V*_max_[S]) + 1/*V*_max_, where v represents the initial velocity, *V*_max_ represents the maximal reaction velocity, and [S] is the substrate (TMB or H_2_O_2_) concentration.

### Bacterial culture and antibacterial experiments in vitro

*H. pylori* was cultured on a Columbia agar plate with 6.5% sterile Defibrinated Sheep Blood and multiple antibiotics (10 μg/mL vancomycin, 5 μg/mL trimethoprim lactate, 5 μg/mL cefsulodin sodium, and 5 μg/mL amphotericin B) under growth conditions (temperature: 37 °C, atmosphere: microaerophilic, 5% O_2_, 10% CO_2_, 85% N_2_) for 3–5 days and then transferred to Dulbecco’s phosphate-buffered saline (DPBS). The as-prepared bacterial solutions (20 μL) and certain amount of PtCo@G@CPB were added into SGF and incubated at 37 °C, 150 rpm, for 30 min. Then the solution was placed on the Columbia agar plate by the spread plate method and cultured for 3–5 days before observing the number of the bacterial colonies.

### Preparation of bacterial samples for TEM/SEM

After incubation with PtCo@G@CPB or PtCo@G in SGF at 37 °C for 30 min, the *H. pylori* solutions (10^6^ CFU) were fixed in 2.5% glutaraldehyde overnight at 4 °C. Then, the above bacterial solutions were centrifuged (425 × *g*, 10 min) and dehydrated through treating with 50, 70, and 90% gradient ethanol for 8 min and 100% ethanol for 15 min. Finally the resulting samples were dropped on carbon film/silicon slice, and the morphology of *H. pylori* was observed on TEM/SEM.

### Live/dead fluorescence staining

After treatment with PtCo@G@CPB, *H. pylori* solutions were incubated with AO and PI at 37 °C for 10 min, followed by washing with DPBS. Then, the live/dead bacterial cells were observed by confocal laser microscopy.

### Cytotoxicity test

Cell viability was evaluated with the cell counting kit-8 (CCK-8). Human gastric epithelial cells (GES-1, 5 × 10^4^ cells per test well) were cultured in RPMI 1640 (Gibco) supplemented with 10% fetal bovine serum and 1% penicillin-streptomycin on the 96-well plate for 24 h at 37 °C. After culture, PtCo@G@CPB (0–250 μg/mL) was added into the cells and incubated for 24 h. Afterwards, the cells were washed with DPBS for three times and 10% CCK-8 solution was added. After incubation in 5% CO_2_ at 37 °C atmosphere for 0.5 h, the absorbance value at 450 nm was measured by a Synergy 2 Multi-Mode Microplate Reader (Bio-Tek, Winooski, VT). The experimental processes were operated at least three times.

### Establish *H. pylori*-infected mouse model

BALB/c female mice 3–4 weeks old (housing conditions, dark/light cycle: 12/12 h, temperature: 20 °C, humidity: about 40%) were purchased from the Hunan SJA Laboratory Animal Co., Ltd (Changsha, China) and used under protocols approved by the Institutional Animal Care and Use Committee of Hunan University. Each BALB/c mouse received 300 μL of 5 × 10^7^ CFU mL^−1^ *H. pylori* in DPBS by oral gavage once a day for 4 days (on day 4, 5, 6, and 7, respectively), and the infection was allowed to develop for 2 weeks. Then, the established *H. pylori*-infected mice (*n* = 6) were sacrificed, and the stomachs were excised from the abdominal cavity. The stomach was cut longitudinally along the greater curvature. The reculture of gastric mucosa and Gram staining of gastric tissue section were used to demonstrate the successful colonization of *H. pylori* in the stomach of mice.

### The retention of PtCo@G@CPB in vivo

The *H. pylori*-infected mice (6–7 weeks) were randomly divided into two groups (*n* = 6) and received DPBS (300 μL) or PtCo@G@CPB (25 mg/kg, 300 μL) by intragastric administration. The groups of mice were administered with DPBS as negative control. After 4 or 24 h of oral administration, the T_2_-weighted phantom imaging of PtCo@G@CPB in mouse stomach was measured by 1.5 T small animal MR scanner (MRI 1.5 T; Shining Global Science and Education Equipment Co., Ltd., Shanghai, China). After measuring T_2_-weighted phantom imaging of PtCo@G@CPB in mice stomach, the mice were sacrificed, and the stomachs were excised and cut open longitudinally along the greater curvature. The gastric tissue sections were prepared by the paraffin-embedded method. The D- and G-band of PtCo@G in the gastric tissue section were detected by Raman spectroscopy (Renishaw, UK) to certify the retention of PtCo@G@CPB in the mouse stomach. In addition, the collected stomach organs of mice were nitrated with Lefort aqua regia (V_HCl_:V_HNO3_ = 1:3). The amounts of Pt in the organs’ nitrifying liquid were determined by inductively coupled plasma series mass spectrometer (Agilent, USA) to further certify the retention of PtCo@G@CPB in the mouse stomach.

### PtCo@G@CPB selective treatment *H. pylori* in vivo

The established *H. pylori*-infected mice were randomly divided into three treatment groups (*n* = 6) to receive DPBS, PtCo@G@CPB (25 mg/kg) or triple therapy OAC (omeprazole, amoxicillin, clarithromycin) by oral administration once daily for 4 consecutive days (on day 1, 2, 3, and 4, respectively). For the triple therapy group, the treatment mice were first administered omeprazole (a proton pump inhibitor) through oral gavage at 400 μmol/kg, followed by a lag time of 30 min before administration of the other two antibiotics, including 28.5 mg/kg of amoxicillin and 14.3 mg/kg of clarithromycin. The groups of mice were treated with DPBS as negative control. Two days (48 h) after the last administration, the three groups of treatment mice were sacrificed, and the stomachs were excised from the abdominal cavity. The stomach was cut longitudinally along the greater curvature, and the gastric tissues were used for bacterial colonization and tissue section staining study. At the same time, the intestine and feces of the mice were also collected. For *H. pylori* colonization, gastric tissue was suspended in 1 mL DPBS and homogenized for bacterial recovery. The homogenate was spotted on Columbia agar plate with 6.5% sterile Defibrinated Sheep Blood and multiple antibiotics (10 μg/mL vancomycin, 5 μg/mL trimethoprim lactate, 5 μg/mL cefsulodin sodium, and 5 μg/mL amphotericin B). Then, the plates were incubated at 37 °C under microaerobic conditions (5% O_2_, 10% CO_2_, 85% N_2_) for 5 days, and the *H. pylori* colonies were enumerated. For the effects of PtCo@G@CPB on the symbiotic bacteria, the bacterial load in the intestine and feces were determined by quantitative real-time PCR using the protocol in ref. ^46,47^. The test results were normalized to intestine/feces weight. The primer sequences for quantitative real-time PCR are listed in Supplementary Table 3.

### The PtCo@G@CPB toxicity in vivo

During treatment, changes in mice weight were recorded daily for 6 consecutive days. Mice received daily gavage of DPBS, PtCo@G@CPB or triple therapy OAC for 4 consecutive days. Mice were sacrificed on the sixth day, and the stomachs were excised. The gastric tissue sections were prepared by the paraffin-embedded method for H&E staining. The inflammation and injury score of stomach were scored by Dr. Hu in a blind manner according to the H&E staining, and the specific scoring criteria used were detailed in the method in ref. ^8^. Inflammation was graded on a 0–3 ordinal scale based on the Sydney System as follows: chronic inflammation (mononuclear cell infiltration independent of lymphoid follicles); grade 0, no inflammation; grade 1, mild inflammation (slight increase in mononuclear cells); grade 2, moderate inflammation (dense, but focal, mononuclear inflammatory cells); and grade 3, severe inflammation (dense and diffuse mononuclear inflammatory cells). The appearance of the mucosa was graded as follows: 0, normal; 1, spotty changes in cellular staining characteristics of some surface epithelial cells in an otherwise normal mucosa (mild injury); 2, more generalized changes and/or disruption of the surface epithelium in several areas (moderate injury); 3, extensive mucosal destruction (severe injury). The heart, liver, spleen, lung, and kidney of mice were gathered and nitrated with Lefort aqua regia (V_HCl_:V_HNO3_ = 1:3). The amounts of PtCo@G@CPB in the heart, liver, spleen, lung, and kidney of mice were determined by measuring Pt content in the organs’ nitrifying liquid with inductively coupled plasma series mass spectrometer (Agilent, USA).

### Statistics and reproducibility

All the quantitative data in each experiment were presented as mean ± SD and *P* values were calculated by the Student’s two-sided *t*-test. All the data in our manuscript were repeated at least three times independently with similar results.

### Reporting summary

Further information on research design is available in the Nature Research Reporting Summary linked to this article.

## Supplementary information

Supplementary Information

Reporting Summary

## Data Availability

All data that support the findings of this study are available within the article and the Supplementary Information or from the corresponding author upon request. Source data are provided with this paper.

## Source data

Source Data
