# Supplementary material for: In vivo activation of pH-responsive oxidase-like graphitic nanozymes for selective killing of Helicobacter pylori
Source: Nat Commun. 2021 Mar 31;12:2002. doi: 10.1038/s41467-021-22286-x (PMC8012368; doi:10.1038/s41467-021-22286-x)
Supplement: Supplementary file 1 — Supplementary Information [file 41467_2021_22286_MOESM1_ESM.pdf]

Supplementary Information

In vivo activation of pH-responsive oxidase-like graphitic nanozymes for selective killing of *Helicobacter pylori*

Lufeng Zhang[1], Liang Zhang[1], Hui Deng[1], Huan Li[2], Wentao Tang[1], Luyao Guan[1], Ye Qiu[1], Michael J Donovan[1], Zhuo Chen[1]* & Weihong Tan[1,3]

[1]Molecular Science and Biomedicine Laboratory, State Key Laboratory of Chemo/Bio-Sensing and Chemometrics, College of Chemistry and Chemical Engineering, College of Biology, Hunan Provincial Key Laboratory of Biomacromolecular Chemical Biology, Hunan University, Changsha, 410082, China

[2]Department of Gastroenterology, the Third Xiangya Hospital of Central South University, Changsha, 410013, China

[3]The Cancer Hospital of the University of Chinese Academy of Sciences, Institute of Basic Medicine and Cancer (IBMC), Chinese Academy of Sciences, Hangzhou, Zhejiang, 310022, China

These authors contributed equally: Lufeng Zhang, Liang Zhang.

*E-mail: zhuochen@hnu.edu.cn

Supplementary Figures

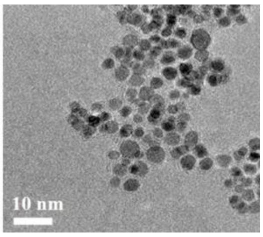

Supplementary Figure 1. TEM image of PtCo@G.

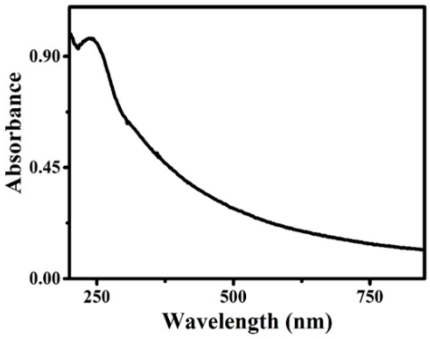

Supplementary Figure 2. UV-Vis absorbance of PtCo@G solution. Source data are provided as a Source Data file.

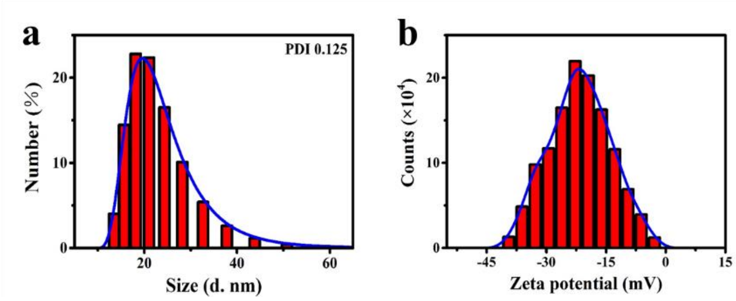

Supplementary Figure 3. The characterization of PtCo@G. (**a**) Hydrodynamic diameter and (**b**) Zeta potential of PtCo@G characterized by dynamic light scattering. Source data are provided as a Source Data file.

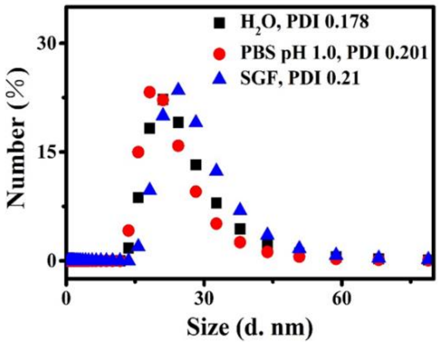

Supplementary Figure 4. Hydrodynamic diameter of PtCo@G in H_2O, PBS (pH 1.0), and SGF after 12 h incubation. Source data are provided as a Source Data file.

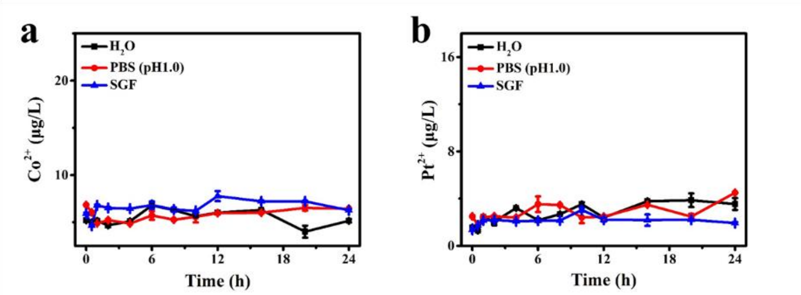

Supplementary Figure 5. The dissolution experiments of PtCo@G. Profiles for (**a**) Co^{2+} and (**b**) Pt^{2+} release from PtCo@G nanocrystals in H_2O, PBS (pH 1.0) and SGF. The data indicate the means and SD from three parallel experiments. Source data are provided as a Source Data file.

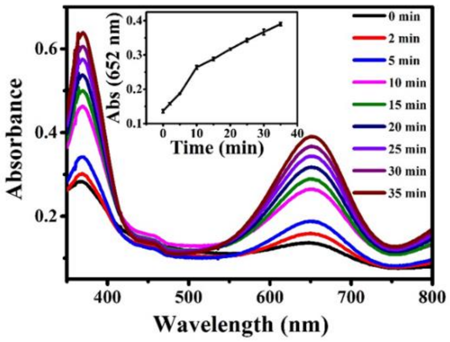

Supplementary Figure 6. Time-dependent absorbance spectra of TMB catalyzed by PtCo@G. Source data are provided as a Source Data file.

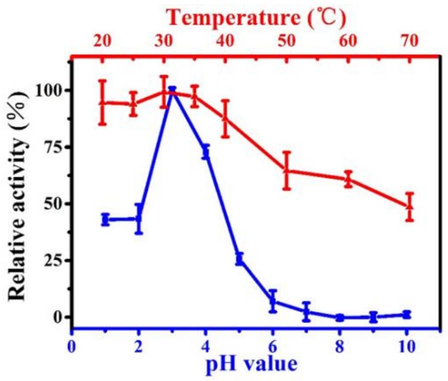

Supplementary Figure 7. The oxidase-like activity of PtCo@G is dependent on pH and temperature. The data indicate the means and SD from three parallel experiments. Source data are provided as a Source Data file.

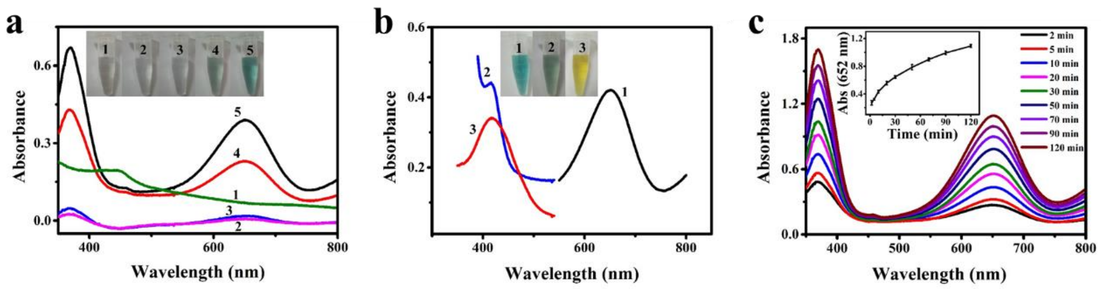

Supplementary Figure 8. Peroxidase-like activity of PtCo@G nanocrystals. (**a**) The UV-Vis absorbance spectra and visual color changes of TMB in different reaction systems: (1) PtCo@G, (2) TMB, (3) TMB+H_2O_2, (4) PtCo@G+TMB, and (5) PtCo@G+TMB+H_2O_2 in a pH 4.0 PBS buffer after 10 min incubation. (**b**) PtCo@G catalyzes oxidation of various peroxidase substrates in the presence of H_2O_2 to produce different color reactions. (1) TMB, (2) ABTS, (3) OPD. Insert images show corresponding visual color changes. (**c**) Time-dependent absorbance spectra of TMB catalyzed by PtCo@G in the presence of H_2O_2. Data are presented as means $\pm$standard deviation (s.d.). Error bars were based on s.d. of three independent experiments. Source data are provided as a Source Data file.

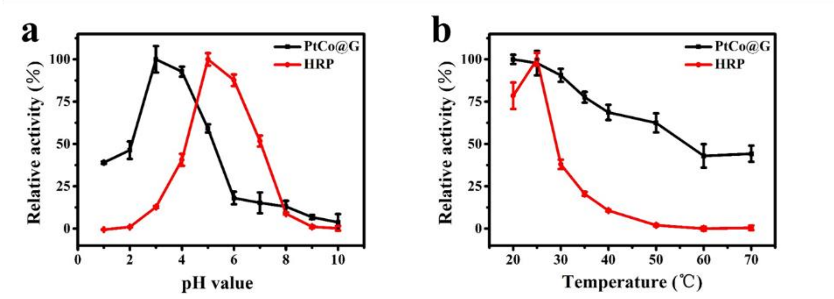

Supplementary Figure 9. Peroxidase-like activity of PtCo@G nanocrystals. (**a**) and (**b**) show that the peroxidase-like activity of PtCo@G is dependent on pH and temperature. Data are presented as means ±s.d. Error bars were taken from three independent experiments. Source data are provided as a Source Data file.

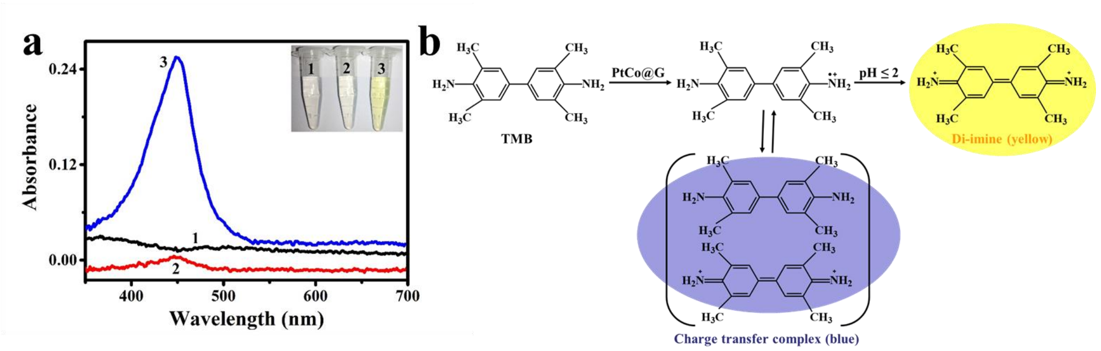

Supplementary Figure 10. The catalytic oxidation of TMB by PtCo@G at strong acidic pH condition. (**a**) Oxidase-like activity of the PtCo@G nanocrystals in PBS (pH 1.0) buffer: (1) PtCo@G, (2) TMB, (3) PtCo@G+TMB. Insert images show corresponding visual color changes. (**b**) The schematic illustration for catalytic oxidation process of TMB by PtCo@G. At weak acidic pH condition, TMB could be oxidized by the oxidase-like PtCo@G to produce blue colored charge transfer complexes. However, the oxidation of TMB in strong acidic pH yielded yellow di-imine products. Source data are provided as a Source Data file.

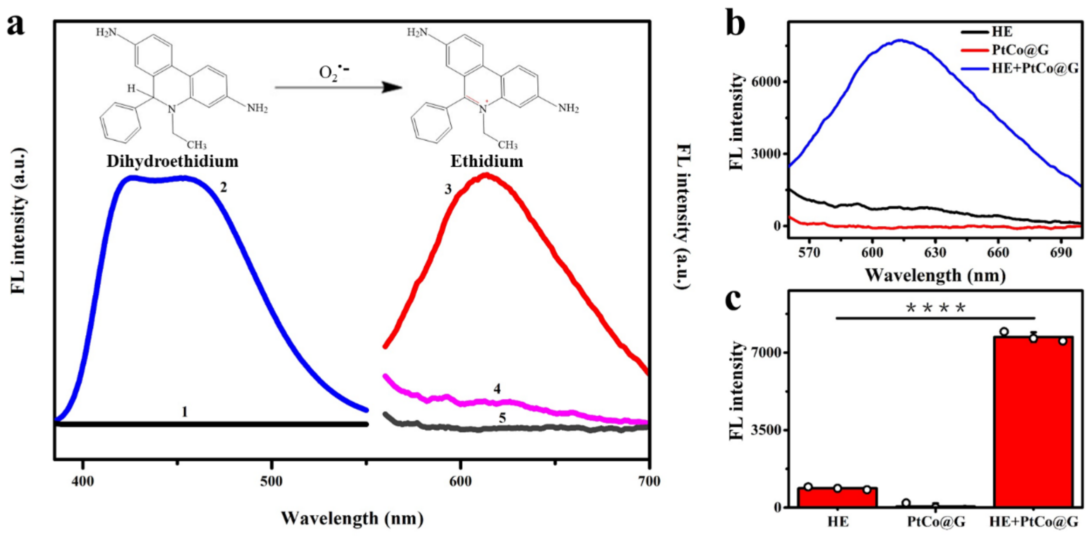

Supplementary Figure 11. The characterization of the superoxide radical ($O_2^{\bullet-}$) generation during the oxidase-like activity of PtCo@G nanocrystals. (**a**) Fluorescence spectra of hydroethidine (HE) in different reaction systems: (1) PtCo@G+HE, (2) HE, (Excitation: 370 nm) (3) PtCo@G+HE, (4) HE, (5) PtCo@G (Excitation: 535 nm) in pH 1.0 PBS buffer after 30 min incubation. Insert shows the reaction of HE as a fluorescence probe for trapping the superoxide radical ($O_2^{\bullet-}$). (**b**) Fluorescence spectra of hydroethidine (HE) before and after the addition of PtCo@G nanocrystals. (**c**) Histograms of FL intensity show the fluorescence product ethidium catalyzed by PtCo@G nanocrystals. *P* values were calculated by the Student's two-sided *t* test: ****$p < 0.0001$. Data are presented as means ± s.d. Error bars were taken from three independent experiments. Source data are provided as a Source Data file.

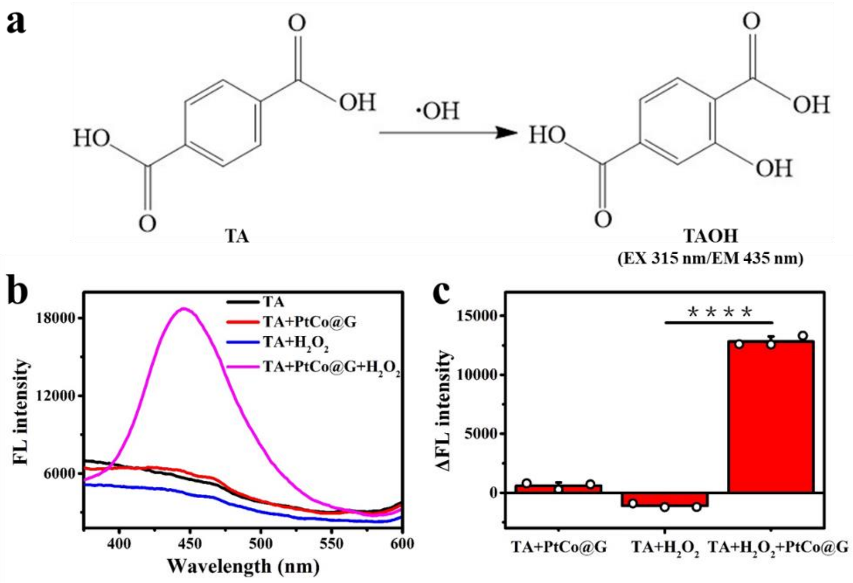

Supplementary Figure 12. The characterization of the hydroxyl radical (•OH) generation during the peroxidase-like activity of PtCo@G nanocrystals. (**a**) The reaction between terephthalic acid (TA) and hydroxyl radical (•OH). The fluorescence product is 2-hydroxy terephthalic acid (TAOH). (**b**) Fluorescence spectra of the PBS (pH 1.0) solution include only TA; TA and PtCo@G; TA and H_2O_2; TA, PtCo@G and H_2O_2. (**c**) Histograms of ΔFL intensity show the fluorescence product, 2-hydroxy terephthalic acid (TAOH) catalyzed by PtCo@G nanocrystals. *P* values were calculated by the Student's two-sided *t* test: ****$p < 0.0001$. Data are presented as means ± s.d. Error bars were taken from three independent experiments. Source data are provided as a Source Data file.

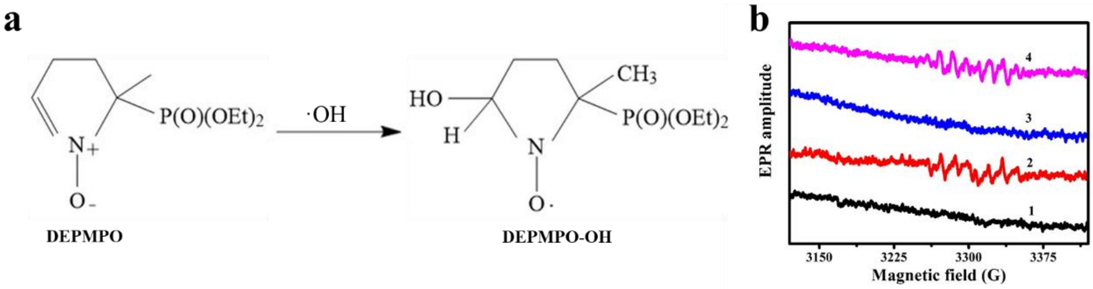

Supplementary Figure 13. The characterization of the hydroxyl radical (•OH) generation during the peroxidase-like activity of PtCo@G nanocrystals. (**a**) The reaction of DEPMPO trapping hydroxyl radical (•OH). (**b**) EPR spectra of DEPMPO-OH obtained by trapping •OH with the spin-trap agent DEPMPO in different reaction systems: (1) Fe^{2+}+H_2O_2, (2) Fe^{2+}+H_2O_2+DEPMPO, (3) PtCo@G+H_2O_2, (4) PtCo@G+H_2O_2+DEPMPO in pH 1.0 PBS buffer. Source data are provided as a Source Data file.

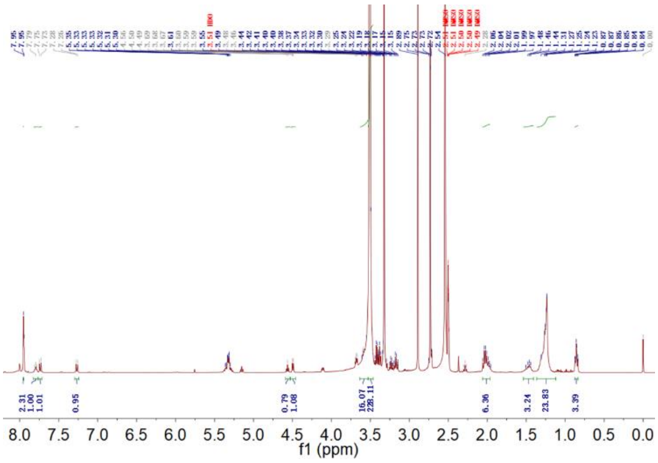

Supplementary Figure 14. ^{1}H-NMR characterization of C_{18}-PEGn-Benzeneboronic acid (CPB) with MQ 400 MHz. Source data are provided as a Source Data file.

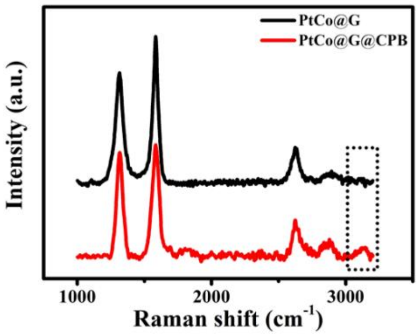

Supplementary Figure 15. Raman spectra characterization of CPB functionalized PtCo@G. Source data are provided as a Source Data file.

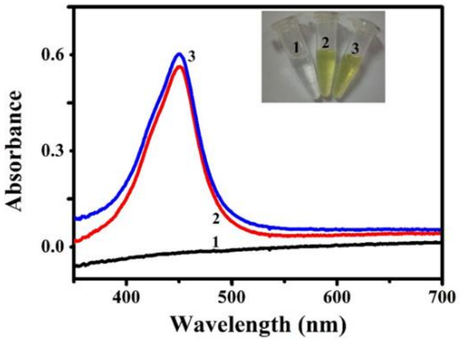

Supplementary Figure 16. The oxidase-like property comparison of PtCo@G and PtCo@G@CPB in SGF: (1) TMB, (2) PtCo@G+TMB, (3) PtCo@G@CPB+TMB. Insert images show corresponding visual color changes. Source data are provided as a Source Data file.

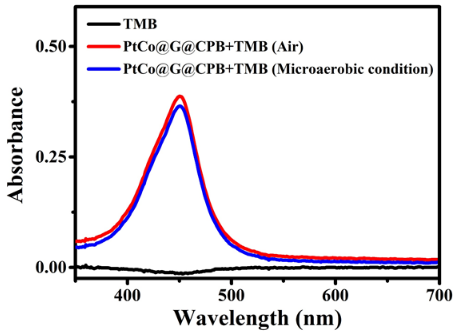

Supplementary Figure 17. The comparison of oxidase-like activity of PtCo@G@CPB in SGF under air and microaerobic atmosphere. Source data are provided as a Source Data file.

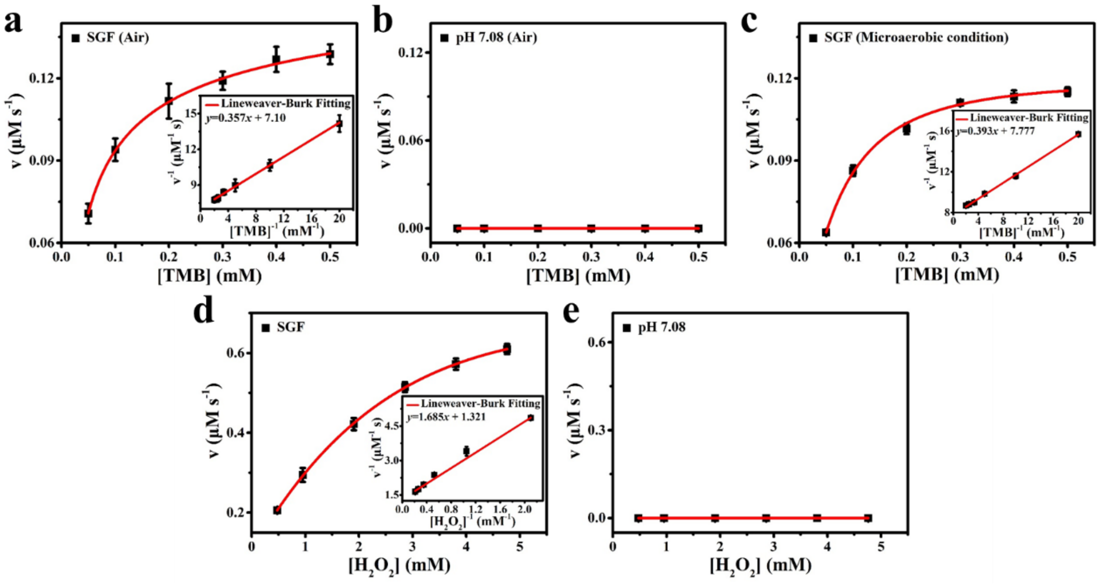

Supplementary Figure 18. The enzyme kinetics of PtCo@G@CPB. Kinetics for (**a**), (**b**), (**c**) oxidase-like and (**d**), (**e**) peroxidase-like activities of PtCo@G@CPB. The data indicate the means and SD from three parallel experiments. Source data are provided as a Source Data file.

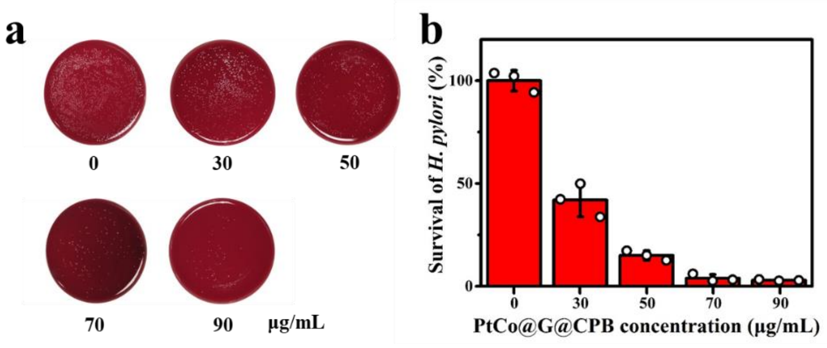

Supplementary Figure 19. The dose-activity test in vitro. (**a**) Photographs of *H. pylori* colonies under treatment with different concentrations of PtCo@G@CPB. (**b**) The antibacterial activity of PtCo@G@CPB at different concentrations against *H. pylori*. The data indicate the means and SD from three parallel experiments. Source data are provided as a Source Data file.

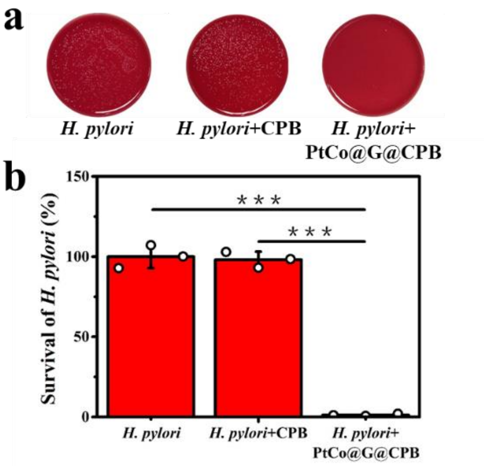

Supplementary Figure 20. The antibacterial activity of PtCo@G@CPB against *H. pylori*. (**a**) Photographs of *H. pylori* colonies under different treatments. (**b**) The bacterial activity of *H. pylori* under different treatments. The amounts of CPB molecules in the experimental groups of *H. pylori* + CPB and *H. pylori* + PtCo@G@CPB (70 μg/mL) are same. *P* values were calculated by the Student's two-sided *t* test: ***$p < 0.001$. The data indicate the means and SD from three parallel experiments. Source data are provided as a Source Data file.

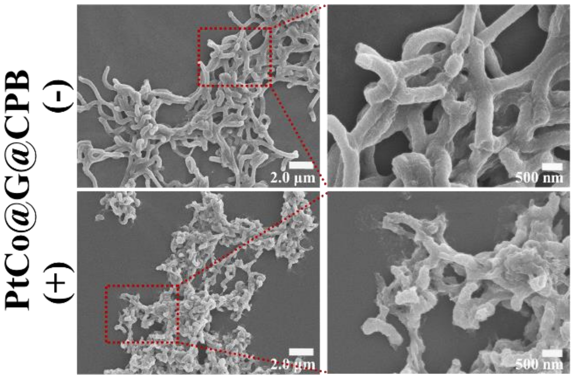

Supplementary Figure 21. SEM images of *H. pylori* treatment without or with PtCo@G@CPB.

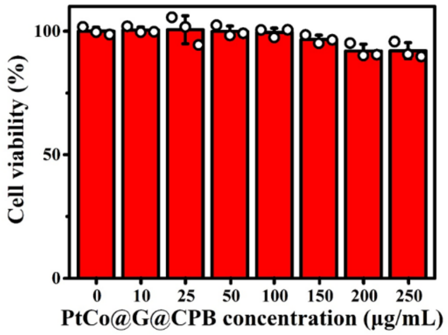

Supplementary Figure 22. Cytotoxicity assay of PtCo@G@CPB. The data indicate the means and SD from three parallel experiments. Source data are provided as a Source Data file.

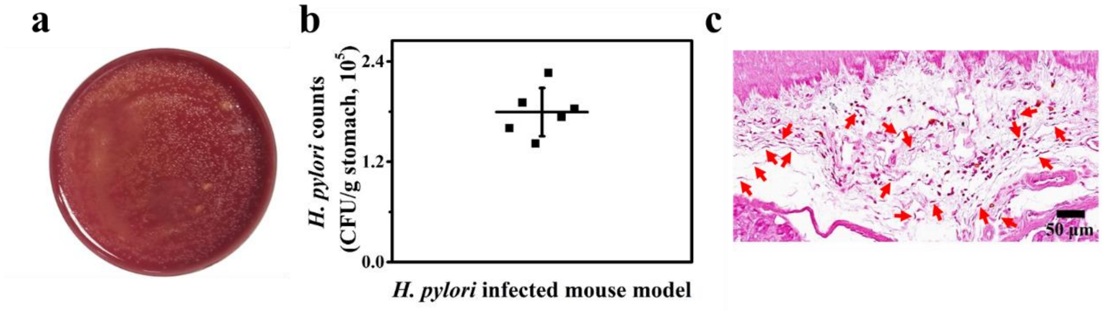

Supplementary Figure 23. Characterization of *H. pylori*-infected mouse model. (**a**) Photographs of *H. pylori* colonies after gastric mucosa reculture. (**b**) Quantification of bacterial burden in the stomach of *H. pylori*-infected mouse. Data are presented as mean ± SD (n=6 biologically independent mice). (**c**) The gram staining of a slice from the gastric mucosa of *H. pylori*-infected mouse. Slice thickness, 5 μm. Red arrows point to bacteria. Source data are provided as a Source Data file.

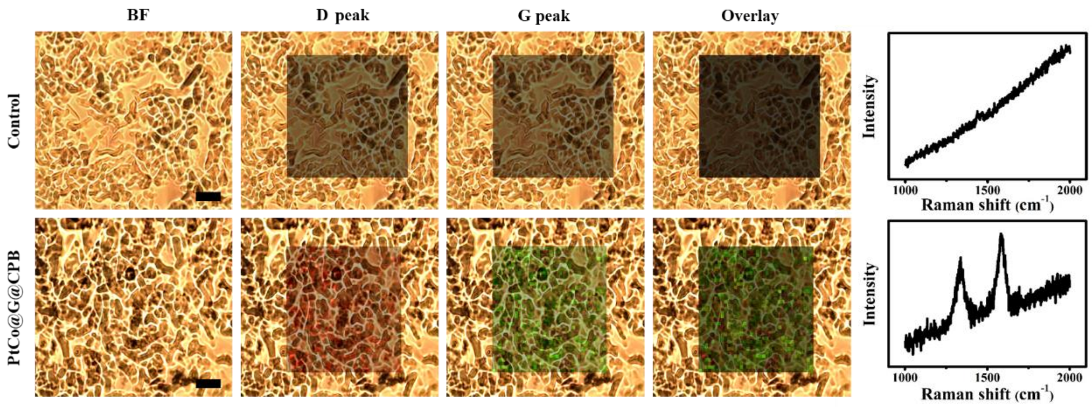

Supplementary Figure 24. Raman imaging and spectra of slice from the gastric mucosa. Scale bar: 20 μm, Slice thickness, 5 μm. Source data are provided as a Source Data file.

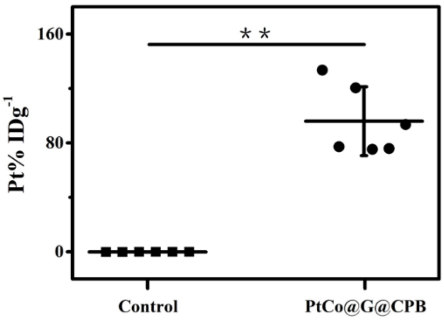

Supplementary Figure 25. The distribution of PtCo@G@CPB in the stomach of *H. pylori*-infected mouse. Data are presented as mean ± SD (n=6 biologically independent mice). *P* values were calculated by the Student's two-sided *t* test: **$p < 0.01$. Source data are provided as a Source Data file.

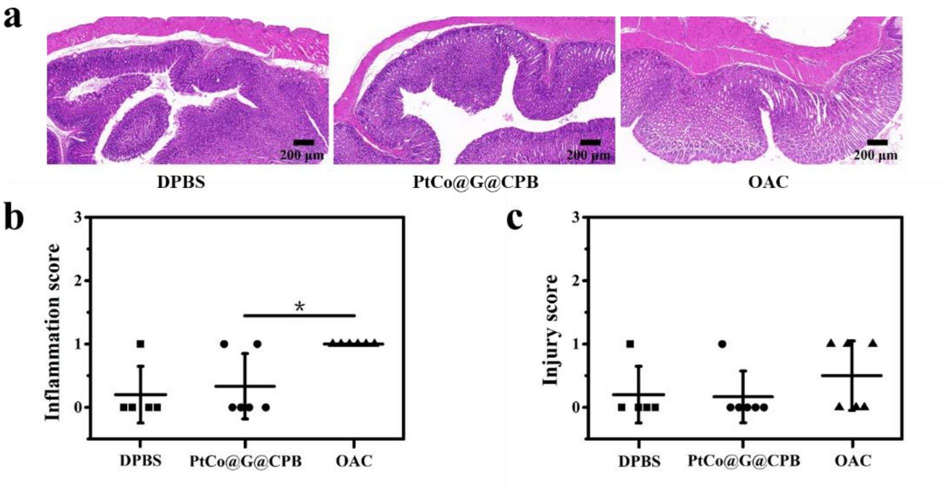

Supplementary Figure 26. The characterization of PtCo@G@CPB toxicity in vivo. (**a**) H&E staining of slice from the gastric mucosa after treatment with DPBS, PtCo@G@CPB and OAC. Slice thickness, 5 μm. The inflammation score (**b**) and injury score (**c**) of gastric mucosa, according to the images of H&E staining. Data are presented as mean ± SD (DPBS: n=5, PtCo@G@CPB and OAC: n=6 biologically independent mice). *P* values were calculated by the Student's two-sided *t* test: **p* = 0.0101. Source data are provided as a Source Data file.

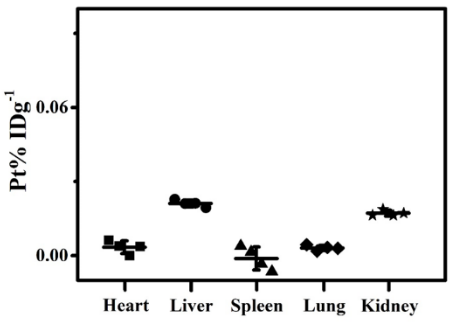

Supplementary Figure 27. The distribution of PtCo@G@CPB in organs after treatment. Data are presented as mean ± SD (n=4 biologically independent mice). Source data are provided as a Source Data file.

Supplementary Tables

Supplementary Table 1. The Michaelis-Menten constant (K_m) and maximal reaction velocity (V_{max}) for the oxidase-like activity of PtCo@G@CPB with TMB as the substrate.

**Catalyst**	**Substrate**	**K_m (mM)**	**V_{max} (μM s^{-1})**	**Reaction condition**
PtCo@G@CPB	TMB	0.0503	0.141	SGF (Air)
PtCo@G@CPB	TMB	0.0505	0.129	SGF (Microaerobic condition)
PtCo@G@CPB	TMB	ND	ND	pH 7.08 (Air)

ND: not detected. The concentration of PtCo@G@CPB was 21.265 μg/mL.

Supplementary Table 2. The Michaelis-Menten constant (K_m) and maximal reaction velocity (V_{max}) for the peroxidase-like activity of PtCo@G@CPB with H_2O_2 as the substrate.

**Catalyst**	**Substrate**	**K_m (mM)**	**V_{max} (μM s^{-1})**	**Reaction condition**
PtCo@G@CPB	H_2O_2	1.276	0.757	SGF
PtCo@G@CPB	H_2O_2	ND	ND	pH 7.08

ND: not detected. The concentration of PtCo@G@CPB was 21.265 μg/mL.

Supplementary Table 3. Sequences of the primers used for quantitative real-time PCR.

**Primer name**	**Primer sequence**	**Note**
Bac16s8F	5-AGAGAGTTTGATCCTGGCTCAG-3	Forward primer
Bac16s338R	5-CATTACCGCGGCTGCTGG-3	Reverse primer
