# Supplementary material for: In vivo activation of pH-responsive oxidase-like graphitic nanozymes for selective killing of Helicobacter pylori
Source: Nat Commun. 2021 Mar 31;12:2002. doi: 10.1038/s41467-021-22286-x (PMC8012368; doi:10.1038/s41467-021-22286-x)
Supplement: Supplementary file 2 — Reporting Summary [file 41467_2021_22286_MOESM2_ESM.pdf]

nature research

Corresponding author(s): Zhuo Chen

Last updated by author(s): Feb 3, 2021

Reporting Summary

Nature Research wishes to improve the reproducibility of the work that we publish. This form provides structure for consistency and transparency in reporting. For further information on Nature Research policies, see our Editorial Policies and the Editorial Policy Checklist.

Please do not complete any field with "not applicable" or n/a. Refer to the help text for what text to use if an item is not relevant to your study.
For final submission: please carefully check your responses for accuracy; you will not be able to make changes later.

Statistics

For all statistical analyses, confirm that the following items are present in the figure legend, table legend, main text, or Methods section.

n/a	Confirmed	
☐	☒	The exact sample size (*n*) for each experimental group/condition, given as a discrete number and unit of measurement
☐	☒	A statement on whether measurements were taken from distinct samples or whether the same sample was measured repeatedly
☐	☒	The statistical test(s) used AND whether they are one- or two-sided<br>*Only common tests should be described solely by name; describe more complex techniques in the Methods section.*
☒	☐	A description of all covariates tested
☐	☒	A description of any assumptions or corrections, such as tests of normality and adjustment for multiple comparisons
☐	☒	A full description of the statistical parameters including central tendency (e.g. means) or other basic estimates (e.g. regression coefficient) AND variation (e.g. standard deviation) or associated estimates of uncertainty (e.g. confidence intervals)
☐	☒	For null hypothesis testing, the test statistic (e.g. *F*, *t*, *r*) with confidence intervals, effect sizes, degrees of freedom and *P* value noted<br>*Give P values as exact values whenever suitable.*
☒	☐	For Bayesian analysis, information on the choice of priors and Markov chain Monte Carlo settings
☒	☐	For hierarchical and complex designs, identification of the appropriate level for tests and full reporting of outcomes
☒	☐	Estimates of effect sizes (e.g. Cohen's *d*, Pearson's *r*), indicating how they were calculated

Our web collection on statistics for biologists contains articles on many of the points above.

Software and code

Policy information about availability of computer code

Data collection	Raman measurements were acquired by WiRE 3.4 software. Ultraviolet-visible spectra were recorded by UVProbe 2.330 software. Hydrodynamic diameters and Zeta potentials were measured by Zetasizer 7.02 software. TEM images were taken with GMS 3 software. SEM images were measured by xT microscope Server 3.0.11 software. Fluorescence spectra were performed by FluorEssence V3.5 software. Electron paramagnetic resonance (EPR) measurements were recorded by ESR 2.0.0.0 software. Bruker the minispec 27010.0 software was used to measure T_2 relaxivity. T_2-weighted MR imaging was taken by Proc_Csharp 1.0.0.0 software. Data from Inductively-coupled plasma mass spectrometry were acquired using IcpMHLauncher 3.01.0005 software.
Data analysis	Origin 9.0 for Raman, UV, Hydrodynamic diameters and Zeta potentials, EPR measurements, T_2 relaxivity and ICP-MS measurements analysis. Image J 2006.02.01 software for T_2-weighted MR imaging analysis. Statistical analysis were done using GraphPad Prism 6 (GraphPad Software, Inc.).

For manuscripts utilizing custom algorithms or software that are central to the research but not yet described in published literature, software must be made available to editors and reviewers. We strongly encourage code deposition in a community repository (e.g. GitHub). See the Nature Research guidelines for submitting code & software for further information.

Data

Policy information about availability of data

All manuscripts must include a data availability statement. This statement should provide the following information, where applicable:
- Accession codes, unique identifiers, or web links for publicly available datasets
- A list of figures that have associated raw data
- A description of any restrictions on data availability

All data that support the findings of this study are available within the article and the Supplementary Information or from the corresponding author upon request. Source data are provided with this paper.

Field-specific reporting

Please select the one below that is the best fit for your research. If you are not sure, read the appropriate sections before making your selection.

☒ Life sciences ☐ Behavioural & social sciences ☐ Ecological, evolutionary & environmental sciences

For a reference copy of the document with all sections, see nature.com/documents/nr-reporting-summary-flat.pdf

Life sciences study design

All studies must disclose on these points even when the disclosure is negative.

Sample size	No specific sample-size calculation was performed during experiments. Adequate sample sizes are generally determined based on extensive laboratory experience and capable of statistical analysis and described in the figure legend.
Data exclusions	No data were excluded from the analyses.
Replication	Each experiment was performed 3-6 independent replications and the exact n-numbers were described in the figure legend.
Randomization	All mice in the study were randomly assigned to the experimental groups. Enough replications and the control groups were carried out in consistent experimental condition.
Blinding	The investigators were not blind to mice allocation during data collection and analysis. All the data of T_2-weighted MR imaging, Raman imaging, Gram staining and Relative 16S rRNA gene level were collected by the instruments directly leaving no room for subjectivity.

Reporting for specific materials, systems and methods

We require information from authors about some types of materials, experimental systems and methods used in many studies. Here, indicate whether each material, system or method listed is relevant to your study. If you are not sure if a list item applies to your research, read the appropriate section before selecting a response.

Materials & experimental systems

n/a	Involved in the study	
☒	☐	Antibodies
☐	☒	Eukaryotic cell lines
☒	☐	Palaeontology and archaeology
☐	☒	Animals and other organisms
☒	☐	Human research participants
☒	☐	Clinical data
☒	☐	Dual use research of concern

Methods

n/a	Involved in the study	
☒	☐	ChIP-seq
☒	☐	Flow cytometry
☒	☐	MRI-based neuroimaging

Eukaryotic cell lines

Policy information about cell lines

Cell line source(s)	The GES-1 cell line used in this study was obtained from iCell Bioscience Inc, Shanghai.
Authentication	Cell lines were authenticated using Short Tandem Repeat (STR) analysis.
Mycoplasma contamination	The cell line used in the study was tested for mycoplasma contamination and the result was negative.
Commonly misidentified lines (See ICLAC register)	No commonly misidentified lines were used.

Animals and other organisms

Policy information about studies involving animals; ARRIVE guidelines recommended for reporting animal research

Laboratory animals	BALB/c female mice 3-4 weeks old (housing conditions, dark/light cycle: 12/12 hours, temperature: 20°C, humidity: about 40%) were used in the study.
Wild animals	The study did not involve wild animals.
Field-collected samples	The study did not involve samples collected from the field.
Ethics oversight	The study protocols were approved by the Institutional Animal Care and Use Committee of Hunan University.

Note that full information on the approval of the study protocol must also be provided in the manuscript.

This checklist template is licensed under a Creative Commons Attribution 4.0 International License, which permits use, sharing, adaptation, distribution and reproduction in any medium or format, as long as you give appropriate credit to the original author(s) and the source, provide a link to the Creative Commons license, and indicate if changes were made. The images or other third party material in this article are included in the article's Creative Commons license, unless indicated otherwise in a credit line to the material. If material is not included in the article's Creative Commons license and your intended use is not permitted by statutory regulation or exceeds the permitted use, you will need to obtain permission directly from the copyright holder. To view a copy of this license, visit http://creativecommons.org/licenses/by/4.0/
